# Strawberry Tongue due to Streptococcal Toxic Shock Syndrome

**DOI:** 10.1002/ccr3.72915

**Published:** 2026-06-08

**Authors:** Hiro Takefuji, Junpei Komagamine, Kei Ota

**Affiliations:** ^1^ Department of Emergency and Critical Care Medicine NHO Tokyo Medical Center Tokyo Japan

**Keywords:** pelvic inflammatory syndrome, strawberry tongue, streptococcal toxic shock syndrome, *Streptococcus pyogenes*

## Abstract

Strawberry tongue is associated with streptococcal toxic shock syndrome (TSS). Streptococcal TSS should be considered as one of the differential diagnoses for patients with shock and strawberry tongue. Our case is the first to report desquamation of strawberry tongue in patients with TSS.

## 1 Case Presentation

**FIGURE 1:**
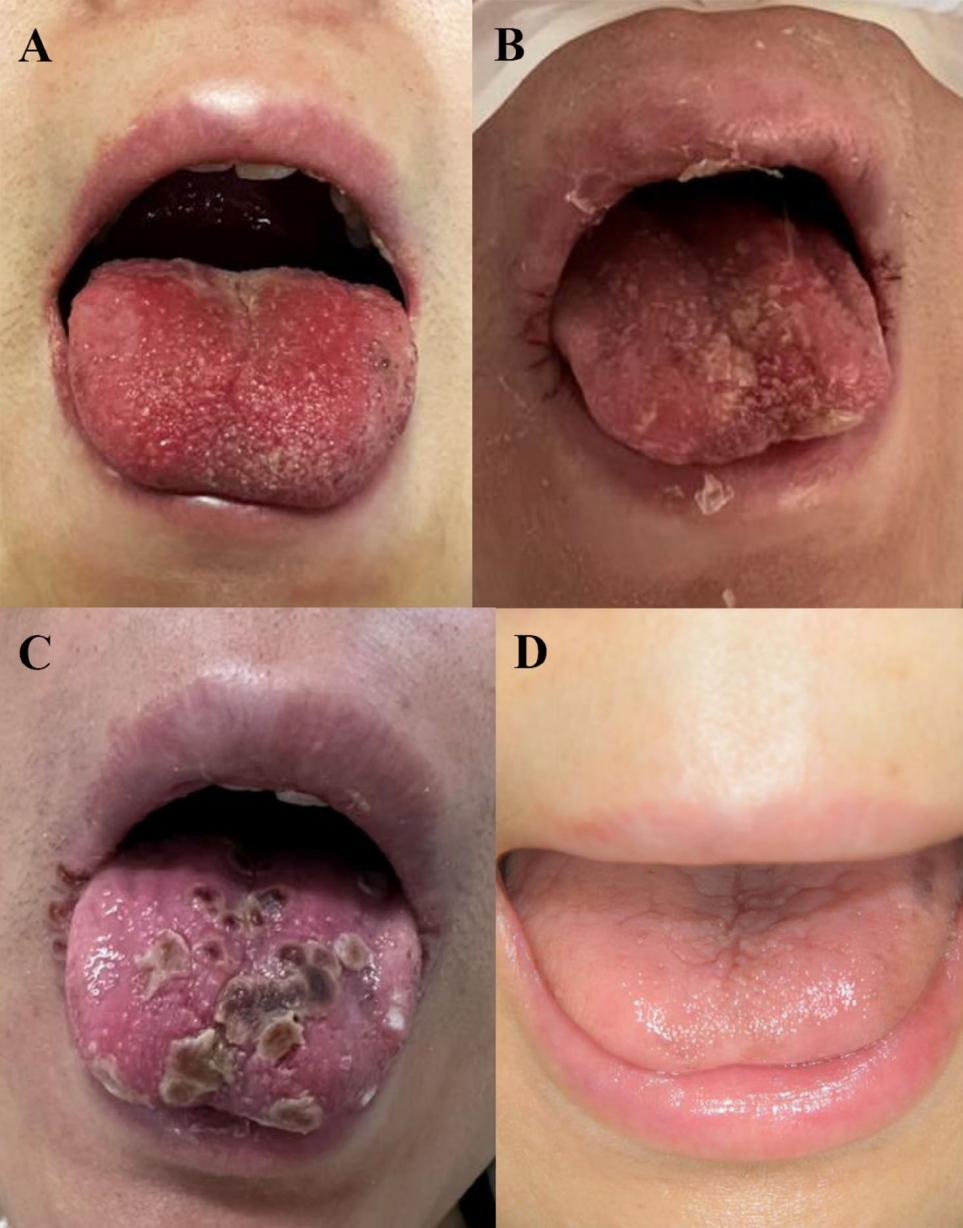
(A) Strawberry tongue was evident on the third hospital day. (B) Crusts appeared on the seventh hospital day. (C) Shallow ulcers developed on the tenth hospital day. (D) The tongue appeared almost normal at discharge.

A 37‐year‐old nulligravid woman with no history of gynecologic disease was admitted to our hospital because of fever and abdominal pain for 2 days. Her medical history was unremarkable, but she had undergone vaginal irrigation multiple times 2 weeks prior. Her vital signs and physical findings were not significant. Abdominal computed tomography revealed thickening of her bowel wall and a small amount of ascites. Acute gastroenteritis was diagnosed, and an intravenous fluid infusion was started. After admission, the patient gradually developed diarrhea and vomiting, and she exhibited hypotension on the third hospital day. Physical examination revealed diffuse erythroderma, strawberry tongue (Figure 1A), and conjunctivitis. A pelvic examination revealed adnexal tenderness. Laboratory tests revealed liver and kidney injuries and elevated C‐reactive protein levels. Vancomycin 1 g SID, minocycline 100 mg BID, clindamycin 600 mg TID, and ceftriaxone 2 g SID were started, and the patient was transferred to the intensive care unit. Although the patient's blood cultures revealed no organisms, culture of her vaginal discharge revealed *Streptococcus pyogenes* . Thus, streptococcal toxic shock syndrome (TSS) due to pelvic inflammatory disease (PID) was diagnosed. Her condition gradually improved with intensive care and laparoscopic abdominal lavage, and antibiotics were de‐escalated to sulbactam/ampicillin 3 g QID. She was ultimately discharged on the 31st hospital day. Crusts had appeared on the patient's tongue on the seventh hospital day (Figure 1B), and shallow ulcers had developed on the tenth hospital day (Figure 1C). However, the patient's tongue showed recovery at discharge (Figure 1D).

## 2 Discussion

Strawberry tongue is characterized by a red swollen tongue with hyperplastic fungiform papillae [1]. It is present in several conditions, such as TSS, Kawasaki disease, scarlet fever, and streptococcal pharyngitis [1]. Notably, strawberry tongue is a well‐known clinical finding associated with streptococcal infections. Nonetheless, there are few reports of strawberry tongue associated with streptococcal TSS, although cases of strawberry tongue caused by staphylococcal TSS have often been reported [2]. Given that streptococcal TSS has higher mortality than staphylococcal TSS [3], streptococcal TSS should also be considered in the differential diagnosis for patients with shock and strawberry tongue.

There are few reports describing the chronological changes in strawberry tongue. Moreover, to our knowledge, there are no previous reports regarding desquamation of strawberry tongue in patients with TSS. Our case indicates the importance of sequential examination of the tongue when TSS is suspected.

## Author Contributions

**Hiro Takefuji:** conceptualization, investigation, project administration, writing – original draft. **Junpei Komagamine:** conceptualization, supervision, writing – review and editing. **Kei Ota:** supervision, writing – review and editing.

## Funding

The authors have nothing to report.

## Ethics Statement

The authors have nothing to report.

## Consent

Written informed consent was obtained from the patient to publish this report in accordance with the journal's patient consent policy.

## Conflicts of Interest

The authors declare no conflicts of interest.

## Data Availability

The authors have nothing to report.
